# A specific phase of transcranial alternating current stimulation at the β frequency boosts repetitive paired-pulse TMS-induced plasticity

**DOI:** 10.1038/s41598-021-92768-x

**Published:** 2021-06-23

**Authors:** Hisato Nakazono, Katsuya Ogata, Akinori Takeda, Emi Yamada, Shinichiro Oka, Shozo Tobimatsu

**Affiliations:** 1 Department of Clinical Neurophysiology, Neurological Institute, Faculty of Medicine, Graduate School of Medical Sciences, Kyushu University, Fukuoka, 812-8582 Japan; 2 Department of Occupational Therapy, Faculty of Medical Science, Fukuoka International University of Health and Welfare, Fukuoka, 814-0001 Japan; 3 Department of Pharmaceutical Sciences, School of Pharmacy at Fukuoka, International University of Health and Welfare, Fukuoka, 831-8501 Japan; 4 Research Center for Brain Communication, Research Institute, Kochi University of Technology, Kochi, 782-8502 Japan; 5 Department of Linguistics, Faculty of Humanities, Kyushu University, Fukuoka, 819-0395 Japan; 6 Department of Physical Therapy, School of Health Sciences at Fukuoka, International University of Health and Welfare, Fukuoka, 831-8501 Japan; 7 Department of Orthoptics, Faculty of Medical Science, Fukuoka International University of Health and Welfare, Fukuoka, 814-0001 Japan

**Keywords:** Neuroscience, Neurology

## Abstract

Transcranial alternating current stimulation (tACS) at 20 Hz (β) has been shown to modulate motor evoked potentials (MEPs) when paired with transcranial magnetic stimulation (TMS) in a phase-dependent manner. Repetitive paired-pulse TMS (rPPS) with I-wave periodicity (1.5 ms) induced short-lived facilitation of MEPs. We hypothesized that tACS would modulate the facilitatory effects of rPPS in a frequency- and phase-dependent manner. To test our hypothesis, we investigated the effects of combined tACS and rPPS. We applied rPPS in combination with peak or trough phase tACS at 10 Hz (α) or β, or sham tACS (rPPS alone). The facilitatory effects of rPPS in the sham condition were temporary and variable among participants. In the β tACS peak condition, significant increases in single-pulse MEPs persisted for over 30 min after the stimulation, and this effect was stable across participants. In contrast, β tACS in the trough condition did not modulate MEPs. Further, α tACS parameters did not affect single-pulse MEPs after the intervention. These results suggest that a rPPS-induced increase in trans-synaptic efficacy could be strengthened depending on the β tACS phase, and that this technique could produce long-lasting plasticity with respect to cortical excitability.

## Introduction

Non-invasive brain stimulation (NIBS) is widely used for experimental and clinical applications, and has been found to modulate cortical excitability via several different protocols. NIBS appears to exert an effect through mechanisms similar to long-term potentiation or depression (LTP or LTD)^1,2^, and likely involves N-methyl-D-asparate receptor activation^1^. However, recent studies have demonstrated high inter- and intra-individual variability in the response to NIBS^3–6^, and the source of this variability is unclear^7^. One possibility is that ongoing brain oscillations may obscure the impact of brain stimulation, producing high variability in the effects of NIBS within an individual^8^. Rhythmic brain oscillations underlie network activities that are generated in specific local and large-scale neuronal networks^9^, and reflect the temporally precise interaction of neural activities^10^. Recently, several research groups proposed tuning NIBS to the amplitude and/or phase of this ongoing oscillatory activity, with the goal of enhancing the efficacy of the stimulation^11^. This idea was validated by Zrenner et al.^12^, who applied repetitive transcranial magnetic stimulation (rTMS) that was synchronized with the specific phase of ongoing sensorimotor α oscillations (i.e., Mu-rhythm) as a closed-loop stimulation. They found that this electroencephalography (EEG)-triggered rTMS technique induced plasticity in primary motor cortex (M1) excitability.

Although implementation of the closed-loop approach was successful, technical challenges remain. The closed-loop set-up requires a real-time signal processing system with a time-resolution on the order of milliseconds because TMS pulses are applied to correspond to the instantaneous oscillatory phase^13^. A combined stimulation with transcranial alternating current stimulation (tACS) is another promising approach. tACS is thought to entrain ongoing oscillations in a frequency-dependent manner^14^. Several studies have investigated the after-effects of combined stimulation theta burst stimulation (TBS) and tACS^15,16^. Goldsworthy et al.^15^ showed that the LTD-like neuroplastic response to continuous TBS (cTBS) was enhanced when the cTBS burst stimulations were delivered with the trough phase of 10 Hz (α) tACS. They inferred that the neuroplastic responses to the combined stimulation were the result of entrainment of endogenous α oscillations in sensorimotor areas by α tACS. Meanwhile, in addition to α oscillations, β oscillations have also been observed in the sensorimotor area. β oscillations have been reported to originate from M1^17,18^ and to relate to the modulation of motor evoked potentials (MEPs)^19^. To examine the causal role of β oscillations in M1 excitability in previous studies, the researchers applied tACS at four different frequencies (20 Hz (β) and controlled frequencies of 5, 10, and 40 Hz) over M1 and recorded MEPs during stimulation. As a result, β tACS increased MEP amplitudes compared with other stimulation conditions^20^. Moreover, recent studies have reported that tuning single-pulse TMS to the specific phases of tACS revealed the phase-specific effects of β tACS on M1 excitability^21–24^. However, combined stimulation has not yet been comprehensively examined with respect to β tACS phase and specific NIBS protocols.

**Figure 1:**
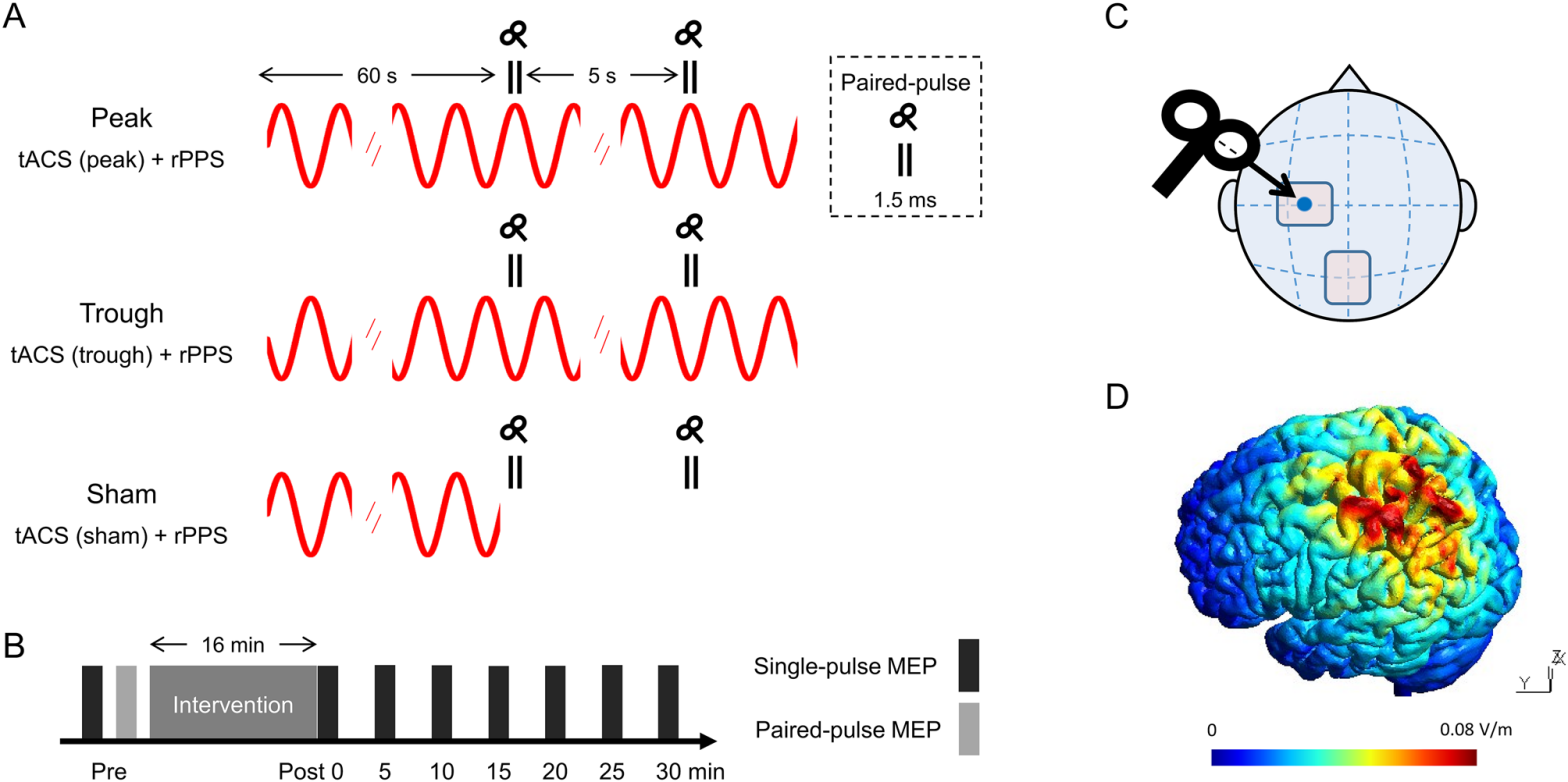
Experimental design. (**A**) Three combined stimulation conditions. Paired-pulse TMS (inter-pulse interval: 1.5 ms) and rPPS was applied according to the different phases of tACS (positive peak phase: peak, negative peak phase: trough), every 5 s for 15 min (180 paired stimuli). rPPS was initiated 60 s after the beginning of tACS. In the sham condition, tACS was applied only for the first 60 s. tACS was applied at 20 Hz (β) and 10 Hz (α) frequencies in Experiments 1 and 2, respectively. (**B**) Time course of our experiments. Data for two baseline conditions, one for single-pulse and one for paired-pulse MEP, were collected before the combined stimulation. The intervention was performed for 16 min, and then single-pulse MEPs were collected every 5 min for up to 30 min after the intervention. (**C**) The electrode montage of tACS and TMS coil configuration. The target electrode was placed on the “hot spot” (blue circle) of the left M1. TMS was applied over the target electrode. (**D**) Simulation of the electrical field for tACS. Abbreviations: MEP, motor evoked potential; rPPS, repetitive paired-pulse TMS; tACS, transcranial alternating current stimulation.

Here, we evaluated whether β tACS could modulate the LTP-like plasticity induced by repetitive paired-pulse TMS (rPPS, also called iTMS^25^) in a phase-dependent manner. In the rPPS protocol, a suprathreshold paired pulse with an inter-pulse interval (IPI) of 1.5 ms was applied repetitively at a rate of 0.2 Hz. rPPS has been suggested to be a safe technique because of the low-frequency rate of stimulation (0.2 Hz)^26,27^. Although previous studies found that rPPS induced LTP-like plasticity, this after-effect was relatively short-lasting (10–15 min)^25,27–29^. We hypothesized that tuning rPPS to a β tACS phase (i.e., peak or trough phase) would induce longer-lasting and more robust effects than would be expected from rPPS alone. Therefore, we performed rPPS that was adjusted to the β tACS phase (Fig. 1A), and evaluated the after-effects by comparing MEPs obtained by single-pulse TMS in Experiment 1 (Fig. 1B). Moreover, we investigated the effects of α tACS on M1 to ascertain the frequency- and phase-dependent effects of tACS in Experiment 2.

## Results

No participants reported any adverse effects during or after any interventions. The baseline MEP amplitudes and TMS intensities in Experiments 1 and 2 are summarized in Table 1. There were no significant differences among the three conditions (all *p* > 0.419). Three participants in Experiment 1 reported a slight flickering sensation during the beginning of the β tACS, which faded away. All participants were unable to identify the differences between the stimulation conditions in Experiments 1 and 2.

**Table 1:** Baseline MEP amplitudes and TMS intensities for single-pulse TMS and paired-pulse TMS in each condition in Experiments 1 and 2.

	Single-TMS MEPs (μV)	Paired-TMS MEPs (μV)	Single-TMS intensity (%MSO)	Paired-TMS intensity (%MSO)
**β tACS (Exp. 1)**				
Peak	819.7 ± 161.7	849.4 ± 152.2	56.4 ± 8.8	46.0 ± 7.6
Trough	845.5 ± 141.6	872.5 ± 118.4	56.7 ± 9.7	46.4 ± 8.1
Sham	816.7 ± 166.9	868.7 ± 132.6	56.5 ± 8.4	46.0 ± 6.7
**α tACS (Exp. 2)**				
Peak	748.8 ± 187.5	793.3 ± 202.5	61.8 ± 10.7	51.5 ± 9.6
Trough	779.5 ± 188.3	780.5 ± 139.9	62.7 ± 10.7	51.1 ± 9.8
Sham	733.8 ± 138	786.9 ± 191.3	62.6 ± 10.4	51.4 ± 10.7

Values are expressed as mean ± SD. %MSO represents the percentage of maximum stimulator output.

### Experiment 1: the effects of rPPS combined with β tACS over M1

**Figure 2:**
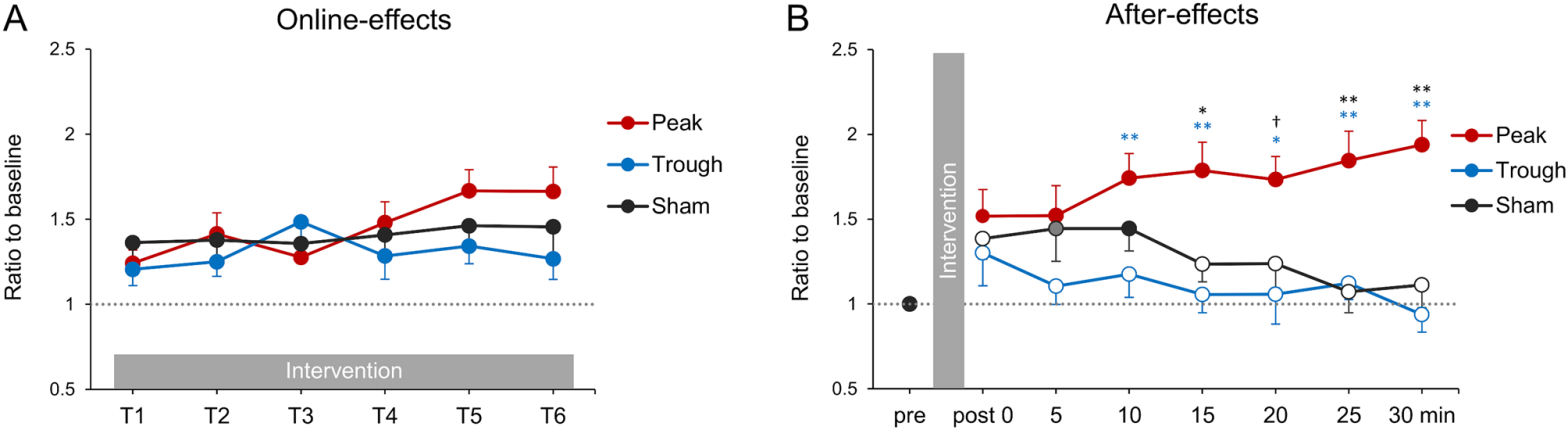
The online- and after-effects of combined stimulation with rPPS and β tACS. (**A**) Changes in the amplitude of paired-pulse MEPs during the intervention (online-effects). The mean MEP amplitudes during the intervention were normalized to paired-pulse MEPs at the baseline. In all three conditions (peak, trough, and sham), MEP amplitudes were increased during the intervention. (**B**) The after-effects of combined stimulation. MEPs were normalized to single-pulse MEPs at the baseline. Filled symbols indicate responses that were significantly different from the baseline (post 5 min in the sham condition, *p* = 0.06; others, *p* < 0.05). In the peak condition, an increase in MEP amplitudes compared with the baseline was observed, and this was sustained for 30 min, while MEPs were enhanced for 10 min after the sham intervention. In contrast, MEPs in the trough condition did not change compared with the baseline. Interestingly, the peak condition resulted in larger enhancements of excitability 15 min after the intervention compared with the other two conditions (peak vs. trough: blue asterisks, peak vs. sham: black asterisks). † = 0.078, * *p* < 0.05, ** *p* < 0.01.

Figure 2A illustrates the serial changes in paired-pulse MEP amplitudes during the intervention (online-effects: T1–T6). MEPs seemed to increase in all conditions, although there were no significant differences among them. A two-way repeated measures analysis of variance (rmANOVA) revealed significant effects of time (*F*[5, 100] = 3.065, *p* = 0.013, η_p_^2^ = 0.133), but no significant effects of the condition (*F*[2, 40] = 1.138, *p* = 0.331, η_p_^2^ = 0.054) or condition × time intervention (*F*[7.7, 154.2] = 1.621, *p* = 0.126, η_p_^2^ = 0.075).

**Figure 3:**
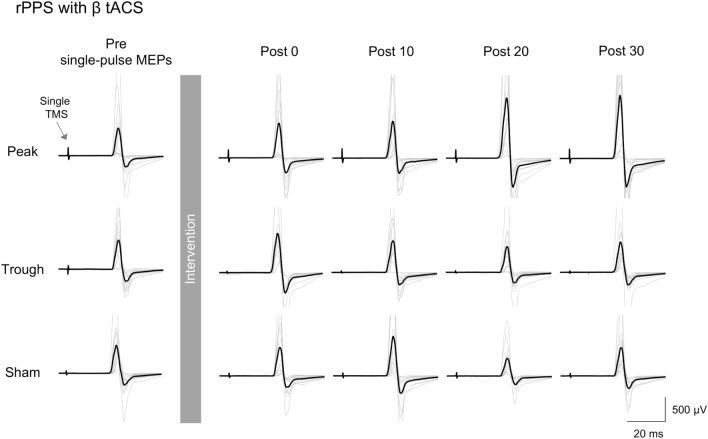
After-effects of combined stimulation with rPPS and β tACS in a representative participant. Single-pulse MEPs illustrated before (Pre; 12 MEP trials) and after the intervention (Post 0, 10, 20, and 30 min; 12 MEP trials, respectively) in the three conditions. The MEPs were averaged (thick lines), and each trial is shown as a thin line. The arrow represents the timing of single-pulse TMS. Only increased MEPs in the peak condition were sustained for over 30 min after the intervention.

Figures 2B and 3 show the time courses of MEP amplitude changes after the intervention (after-effects). We found that the peak stimulation condition increased MEPs, which persisted for over 30 min after the stimulation, while MEP amplitudes were temporarily enhanced in the sham condition (Fig. 2B). By contrast, stimulation in the trough condition did not change M1 excitability. A two-way rmANOVA revealed significant effects of condition (*F*[2, 40] = 13.403, *p* < 0.001, η_p_^2^ = 0.401) and a significant interaction between condition and time (*F*[10.8, 215.1] = 1.93, *p* = 0.038, η_p_^2^ = 0.088), but no significant effect of time (*F*[6, 120] = 0.407, *p* = 0.873, η_p_^2^ = 0.02). Post-hoc analysis revealed that MEP amplitudes were significantly larger in the peak stimulation condition than in the trough and sham conditions (peak vs. sham: post 0–10: *p* > 0.437, post 15: *p* = 0.017, post 20: *p* = 0.078, post 25–30: *p* < 0.01; peak vs. trough: post 0–5: *p* > 0.165, post 10–15: *p* < 0.01, post 20: *p* = 0.015, post 25–30: *p* < 0.01; sham vs. trough: post 0–30: *p* > 0.297). Comparing the baseline with the peak stimulation condition, a one-way rmANOVA revealed a significant effect of time (*F*[7, 140] = 5.879, *p* < 0.001, η_p_^2^ = 0.227). Post-hoc analysis showed a significant increase in MEP amplitudes that persisted for 30 min after the stimulation compared with the baseline condition (post 0–30: *p* < 0.019). In the sham condition, a one-way rmANOVA revealed a significant effect of time (*F*[7, 140] = 2.226, *p* = 0.036, η_p_^2^ = 0.1), and post-hoc analysis revealed that MEP amplitudes were increased for 10 min after the intervention (post 0: *p* = 0.132; post 5: *p* = 0.061; post 10: *p* = 0.039; post15–30: *p* > 0.691). However, a one-way rmANOVA did not reveal a significant effect of time in the trough condition (*F*[7, 140] = 1.126, *p* = 0.35, η_p_^2^ = 0.053).

### Experiment 2: the effects of rPPS combined with α tACS over M1

**Figure 4:**
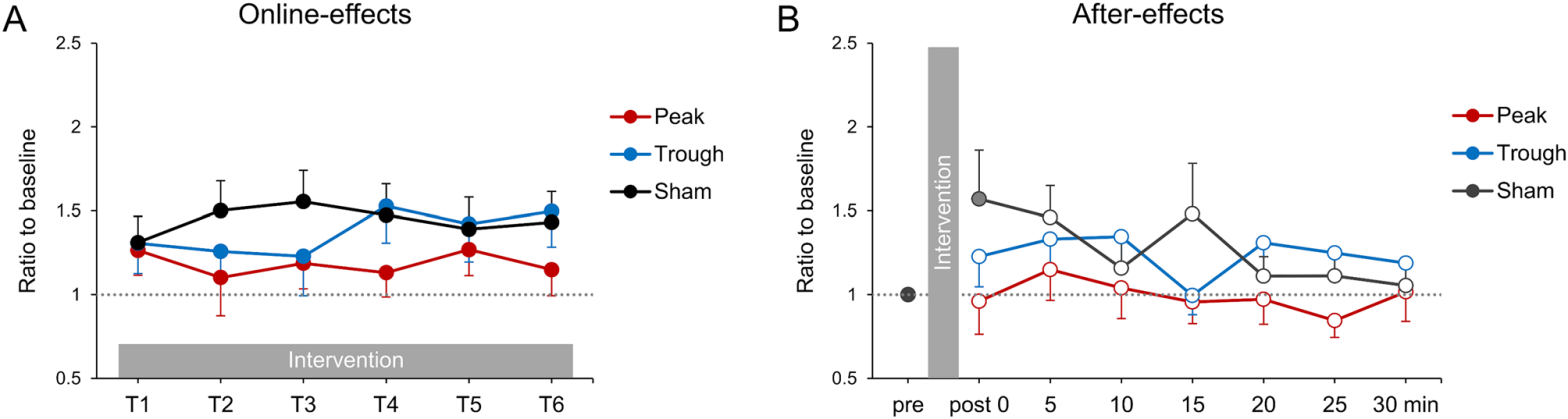
The online- and after-effects of combined stimulation with rPPS and α tACS. (**A**) The online-effects of combined stimulation with rPPS and α tACS on paired-pulse MEPs. The mean MEP amplitudes during the intervention were normalized to paired-pulse MEPs at the baseline. There was no significant difference between conditions. (**B**) The after-effects of combined stimulation with rPPS and α tACS on single-pulse MEPs. MEPs were normalized to single-pulse MEPs at the baseline. There were no significant differences among the three conditions. Compared with the baseline, we observed a tendency toward higher MEP amplitudes in the sham condition, but only immediately after the stimulation (filled symbol in the sham condition, *p* = 0.053).

In terms of online-effects, we found no significant effects of condition (*F*[2, 30] = 0.623, *p* = 0.543, η_p_^2^ = 0.04), time (*F*[3.6, 53.4] = 0.437, *p* = 0.76, η_p_^2^ = 0.028), or the condition × time interaction (*F*[10, 150] = 1.685, *p* = 0.089, η_p_^2^ = 0.101) (Fig. 4A).

We found no significant differences in the after-effects among the three stimulus conditions (peak, trough, and sham) (Fig. 4B). In brief, we found no significant effects of condition (*F*[2, 30] = 1.851, *p* = 0.175, η_p_^2^ = 0.11), time (*F*[6, 90] = 1.303, *p* = 0.264, η_p_^2^ = 0.08), or the condition × time interaction (*F*[6.2, 93] = 1.255, *p* = 0.285, η_p_^2^ = 0.077) (Fig. 4B). Comparing the baseline with the sham condition, a one-way rmANOVA revealed a significant effect of time (*F*[7, 105] = 2.228, *p* = 0.038, η_p_^2^ = 0.129). A post-hoc analysis revealed a tendency toward higher MEP amplitudes in the sham condition compared with those at baseline, but this was only the case immediately after stimulation (post 0: *p* = 0.053; post 5–30: *p* > 0.161). In contrast, a one-way rmANOVA did not reveal a significant effect of time in the peak and trough conditions (*p* > 0.314).

### Variability of effects in Experiments 1 and 2

**Figure 5:**
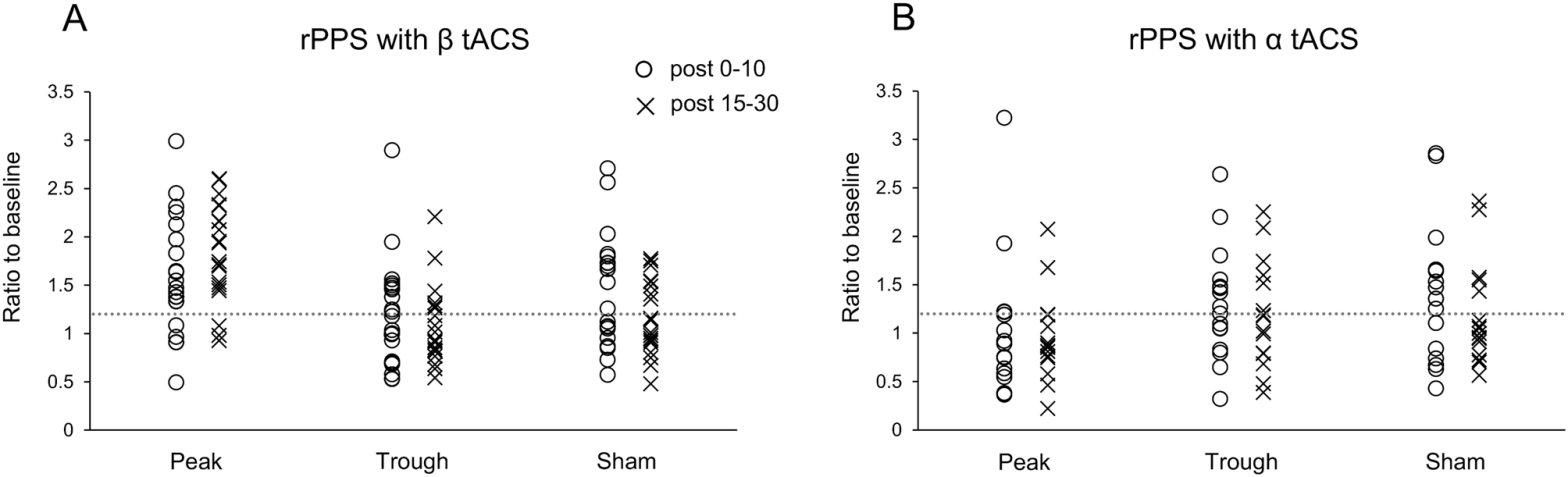
The differential after-effects of each condition on each individual in Experiments 1 (**A**) and 2 (B). The normalized post-intervention MEP amplitudes were averaged (post 0–10 min, post 15–30 min). Dotted lines represent the threshold for facilitatory responses. In the rPPS combined with β tACS condition, excitatory responses under the peak condition were evident in most participants, while those during the trough condition were less marked (**A**). In contrast, we observed no excitatory responses in most participants when rPPS was combined with the α tACS peak phase (**B**). Although MEP amplitudes increased in the sham condition, there was high inter-individual variability in the excitatory responses in Experiments 1 and 2 (**A**, **B**).

Figure 5 shows the individual responses to the different stimulation conditions in Experiments 1 and 2. The after-effects for each participant in each condition were averaged from post 0 to post 10 min, and from post 15 to post 30 min, termed “post 0–10” and “post 15–30”, respectively. Participants were classified as exhibiting an “excitatory response” for mean after-effects (i.e., post 0–10 or post 15–30) > 1.2 or “no response” for mean after-effects < 1.2, according to a previous study^30^. The peak stimulation condition of β tACS elicited an excitatory response in most participants, while the after-effects in other conditions were variable among individuals (excitatory response rate, peak condition: post 0–10 min, 76%; post 15–30 min, 86%; excitatory response rate, trough condition: post 0–10 min, 48%; post 15–30 min, 29%; excitatory response rate, sham condition, post 0–10 min, 52%; post 15–30 min, 38%; Fig. 5A). In Experiment 2, the peak stimulation condition of α tACS induced no excitatory response in most participants, while the after-effects in the other conditions were variable between individuals (excitatory response rate, peak condition: post 0–10 min, 25%; post 15–30 min, 13%; excitatory response rate, trough condition: post 0–10 min, 56%; post 15–30 min, 38%; excitatory response rate, sham condition: post 0–10 min, 63%; post 15–30 min, 31%; Fig. 5B).

## Discussion

We investigated the effects of combined stimulation with tACS and rPPS on M1 excitability. Although rPPS alone induced a short-term increase in MEP amplitudes after the stimulation, rPPS combined with the peak phase of β tACS (i.e., peak condition) prolonged the enhancement of rPPS responses for at least 30 min after the intervention. In contrast, stimulation in the β tACS trough condition did not change M1 excitability. Moreover, α tACS did not have significant phase-dependent effects on M1 in either the peak or trough condition. These results indicate that β tACS over M1 modulates the effects of rPPS on M1 in a frequency- and phase-dependent manner.

### Spike-timing dependent plasticity and rPPS

Previous studies revealed that rPPS increased MEP amplitudes for 10–15 min after stimulation^25,28^. Thus, the after-effects of rPPS do not appear to be long-lasting. For the sham condition (i.e., rPPS alone) in Experiment 1, the time course of the facilitatory effects was similar to that observed in previous studies^25,28^. Single-pulse TMS applied to the M1 has been found to evoke a short series of descending volleys at a periodicity of around 1.5 ms, known as indirect waves (I-waves)^31^. These volleys result from trans-synaptic activation of corticospinal neurons via excitatory and inhibitory neurons^32^. Paired-pulse TMS at a periodicity of about 1.5 ms can produce several peaks in the IPI corresponding to I-wave dynamics, through a process known as short-interval intracortical facilitation (SICF)^33,34^. SICF is thought to result from the temporal summation of the second TMS pulse and the excitatory post-synaptic potentials (EPSPs) induced by the first TMS pulse^35^. An rPPS with an IPI of 1.5 ms could increase synaptic efficacy at the cortical level, which in turn could induce the enhancement of M1 excitability after the stimulation^2,25,29^. Indeed, rPPS increased SICF amplitudes^36^, and did not facilitate the MEP elicited by brainstem electrical stimulation^28^ or F-waves^25^ after stimulation. This intervention could be considered an analogue of spike-timing dependent plasticity (STDP), in which the direction of synaptic efficacy depends on the precise timing of pre- and post-synaptic neuronal activity^2^. However, epidural recordings showed that MEP facilitation at rPPS was accompanied by only a slight increase in the amplitude of descending spinal cord volleys^37^. These findings suggest that rPPS might modulate the neural circuits of broad areas connected to pyramidal tract neurons^1,32^.

### Phase-specific effects of β tACS

In the present study protocol, when rPPS was combined with 90° phase (peak) tACS, we found prolonged after-effects of rPPS, and the excitatory responses elicited by rPPS (excitatory response rate: 76%–86%) were more consistent than when rPPS was applied alone (i.e., the sham condition). However, we did not observe significant modulation of MEP amplitudes after the intervention in the β tACS trough phase, or in the α tACS peak and trough phase conditions. How should these frequency- and phase-specific effects of tACS on M1 be interpreted? It has been suggested that tACS entrains ongoing oscillations in a frequency-dependent manner, which indicates that there exists a causal link between brain oscillations and distinct functions^38,39^. In the sensorimotor area, spontaneous oscillations such as those in the α and β frequency bands are observed. In particular, β oscillations are reported to originate from M1^17,18^, and have been associated with various motor functions (e.g., changes in oscillatory power related to movement, corticomuscular synchronization during isometric muscle contraction)^17,40,41^. Previously, the power and phase of β oscillations in M1 were found to significantly influence MEP amplitudes^42,43^. In tACS studies, β tACS over M1 increased MEP amplitudes during stimulation compared with tACS at other frequencies (i.e., 5, 10, and 40 Hz)^20^. In addition, recent studies have shown that β tACS induces phase-dependent modulation of MEPs^21–24^. However, in these studies, the largest MEP amplitudes were observed at the peak^22^, rising flank^23,24^, or trough^21^ phases when single-pulse TMS was adjusted to the phase of β tACS. These inconsistent findings among previous studies may be the result of differences in experimental parameters (e.g., intensity, electrode size, and montage). Moreover, cumulative effects of single-pulse TMS with β tACS were observed, and these were TMS intensity-dependent^44^. This means that the intervention duration and TMS intensity may influence the phase effects of β tACS on single-pulse TMS. To avoid these unfavorable effects in our previous study, we adopted a short intervention duration (104 s) and the TMS timing was adjusted to the same phase in each trial^22^. In accordance with our previous study, the peak phase of β tACS increased single-pulse MEP amplitudes. However, this was not the case for α tACS^22^. These results are in line with the phase- and frequency-dependent after-effects of β tACS at the 90° (peak) phase. On the basis of the long-lasting after-effects in the β tACS peak condition, synaptic input during paired-pulse TMS is likely to be most effective when it is precisely timed with the neural circuit activity connected to pyramidal neurons via oscillatory synchronization by β tACS.

In terms of the physiological mechanisms underlying the effect of tACS, animal and modelling studies have indicated that an alternating current (AC) field set at the frequency of endogenous oscillations affects neural spike timing, but not firing rate, in a phase-dependent manner^45–47^. Interestingly, β oscillations have been observed in local field potentials in the sensorimotor area of monkeys^48^, and cortical neurons in M1 tend to fire at the β range^49–51^. Therefore, combined stimulation with rPPS and β tACS likely promotes the potentiation of synaptic strength when the I-wave dynamics induced by rPPS coincide with a specific phase at which the entrained M1 is prone to fire. In line with this assumption, a previous animal study revealed that when EPSPs coincided with the peak phase of membrane potential oscillations imposed by AC fields, LTP could be induced^52^. The researchers also showed that spikes with trough phase pairing led to LTD as phase-dependent plasticity^52^. In the present study, the β tACS trough phase condition did not generate inhibitory effects, but abolished the facilitatory effects of rPPS. Thus, the trough phase of β tACS may partially prevent I-wave interaction associated with rPPS.

### NIBS combined with tACS

A previous study that used combined stimulation with tACS and intermittent TBS (iTBS) revealed that the facilitatory effects of iTBS were augmented by γ tACS but not by β tACS^16^. However, the bursts of iTBS were not adjusted to the tACS phase (i.e., phase-unrelated combined stimulation)^16^. One other study investigated the after-effects of combined stimulation with cTBS and α tACS phases^15^. The authors found that the inhibitory neuroplastic response to cTBS was enhanced by the trough phase α tACS. α oscillations have been suggested to reflect the functional inhibition of task-irrelevant brain regions^53^. The inhibitory effect of α tACS may be explained by the functional role of α oscillations^15^. In the current study, rPPS combined with α tACS did not significantly modulate M1 excitability, and most participants showed no excitatory responses to rPPS in the α tACS peak condition only (Fig. 5B). Similarly, our previous study showed that α tACS tended to decrease single-pulse MEPs at the peak phase during stimulation, this was not significant^22^. Although the effective phase of α tACS is different from that in combined stimulation with cTBS, the weak effects of the α tACS peak phase combined with rPPS may reflect the inhibitory effects of α oscillations. Taken together, these data indicate that the effective frequency or phase of tACS in a combined stimulation paradigm is dependent not only on the direction of neuronal plasticity (LTP- or LTD-like plasticity) but also TMS parameters (e.g., paired-pulse or burst TMS). Indeed, a recent animal study showed that the effects of tACS phase on cortical excitability were inverted depending on the parameters (i.e., frequency and duration) of the pulse-train when tACS was combined with suprathreshold pulse-train stimulation^54^.

### Limitations

The present study has several limitations. First, we did not adopt conditions with α and β tACS alone because we could not blind the participants to the stimulus conditions. Previous studies have revealed that β tACS with a higher stimulus intensity increased MEP amplitudes after stimulation^55,56^. However, a recent meta-analysis showed that the after-effects of β tACS were dependent on tACS intensity. Thus, it is possible that β tACS with a lower intensity (≤ 1 mA) is insufficient to produce after-effects^57^. Indeed, β tACS in a study with similar stimulus conditions (the same intensity and electrode montage) to those in our study induced no after-effects on MEPs^58^. We also demonstrated that α and β tACS at an intensity of 1 mA did not induce long-lasting after-effects on M1 cortical excitability^59^. Thus, the phase-dependent effects cannot be explained by the simple add-on effects of β tACS on rPPS. Second, our study had a single-blind design. We used a single-blind design because we needed to monitor the precise timing between rPPS paired-pulses and the tACS phase. Thus, we were unable to use a double-blind design. Third, in contrast to Experiment 1, we found no significant effects of rPPS alone in the sham condition in Experiment 2. Using stimulus conditions that were similar to those in Experiment 2 in the present study, Fitzgerald et al.^60^ found no facilitatory effects after rPPS. Recent studies have demonstrated high inter-individual variability in the response to NIBS (e.g., tDCS and TBS)^3–6^. Indeed, approximately half of the participants in Experiments 1 and 2 exhibited an excitatory response to rPPS alone (i.e., the sham condition), while this was not the case for the other half (the rate of excitatory responses post 0–10 min: Experiment 1: 52%; Experiment 2: 63%). Thus, the results of the sham condition can be explained by the inter-individual variability.

## Conclusion

We found that rPPS applied with the peak phase of β tACS produced a long-lasting after-effect, while the application of β tACS at the trough phase and α tACS at the peak and trough phases did not induce facilitatory effects. Moreover, combined stimulation with rPPS and β tACS at the peak phase decreased inter-individual variability in responses to rPPS. These results suggest that tuning rPPS to the β tACS-phase augments trans-synaptic efficacy by entraining oscillatory brain activities and that this approach could be a novel combined stimulation technique for inducing long-lasting excitability in M1.

## Materials and methods

### Participants

Thirty-seven participants (17 women; mean age ± standard deviation (SD): 23.2 ± 4.5 years old) participated in this study. None of the participants had any history of neurological, psychiatric, or other medical problems. All participants were right-handed, according to the Edinburgh handedness inventory. Written informed consent was obtained from each participant in accordance with the Declaration of Helsinki. This study was approved by the Ethics Committee of Kyushu University and the Ethics Committee of the International University of Health and Welfare. Twenty-one participants (11 women; 24.3 ± 5.7 years old) took part in Experiment 1. In Experiment 2, one participant was excluded because of artifacts in tACS recording data, and data from 16 participants (6 women; 22 ± 2.1 years old; one had also participated in Experiment 1) were included in the analysis. These sample sizes were chosen on the basis of a recent systematic review, which reported that the sample sizes of existing rPPS studies ranged from 6 to 16 participants^27^.

### Motor evoked potentials (MEPs)

Participants were comfortably seated on a chair. Single-pulse TMS was delivered using a monophasic Magstim 200 stimulator (Magstim Co., Whitland, UK) connected to a 70 mm figure-of-eight coil. The coil was held at 45° to the sagittal plane with the handle pointing posterior to induce a posterior-anterior current across the hand area of the left M1. The motor hot spot for the right first dorsal interosseous muscle (FDI) was determined as the site where TMS applied at a slightly suprathreshold intensity elicited the maximum stable MEP responses at rest. This position was identified first to determine the tACS electrode position over the left M1 and then again after the tACS electrodes had been fixed using a support bandage. This site was marked over the support bandage to enable repositioning of the coil. TMS was applied over the tACS electrode overlying the left M1, and the TMS intensity was adjusted to elicit a peak-to-peak MEP amplitude of 500–1000 µV in the relaxed muscle. The intensity was kept constant throughout the experiments. TMS was applied with an inter-stimulus interval ranging from 5 to 7 s. Muscle relaxation was maintained online via visual feedback of electromyographic (EMG) activity. We excluded trials with background EMG activity based on visual inspection in a 200 ms time window preceding the TMS pulse. The average percentage of rejected trials for each participant was 2.4% for Experiment 1 and 3% for Experiment 2.

MEPs were recorded through surface electrodes placed on the right FDI in a belly-tendon derivation. The EMG signals were amplified using the Neuropack 8 system (Nihon Kohden, Tokyo, Japan) with a band-pass filter of 10 Hz–2 kHz, digitized at a sampling rate of 10 kHz and stored in a computer using signal processing software (Multiscope PSTH, Medical Try System, Tokyo, Japan) for offline analysis. The analysis period was 500 ms in length, beginning 250 ms before TMS.

### Repetitive paired-pulse transcranial magnetic stimulation (rPPS)

Paired-pulse TMS was delivered using two Magstim 200 stimulators connected to a Bistim module (Magstim Co., Whitland, UK). Paired stimuli of equal strength were applied with an IPI of 1.5 ms, every 5 s for 15 min (180 paired stimuli)^29,36,61,62^. The stimulus intensity was set to elicit MEP amplitudes of 500–1000 µV when delivered as a pair with an IPI of 1.5 ms.

### Transcranial alternating current stimulation (tACS)

tACS was delivered using a battery-driven current stimulator (DC Stimulator-Plus, NeuroConn GmbH, Ilmenau, Germany) attached to two self-adhesive electrodes (PALS electrodes, Axelgaard Manufacturing Co., Ltd., Fallbrook, CA). The stimulation waveform was sinusoidal and without DC offset. Stimulation was applied at 20 Hz (α) and 10 Hz (β) in Experiments 1 and 2, respectively. tACS was applied at 1 mA (peak-to-peak) for 16 min, with 5 s ramp up and ramp down periods. The target tACS electrode (5 × 7 cm) was placed over the “hot spot” of the left M1 as determined by TMS, whereas the reference electrode (5 × 7 cm) was placed on the midline parietal region (Pz; International 10–20 system) (Fig. 1C). These electrode positions were adopted according to previous studies^21,22^ that demonstrated frequency- and phase-specific tACS effects on M1 excitability. The skin on the scalp around the left M1 and Pz area was cleaned with alcohol, and electrode gel (Gelaid, Nihon Kohden, Tokyo, Japan) was applied to reduce the electrode impedance. The impedance was kept below 5 kΩ during the experiment. The tACS electrodes were affixed using a support bandage.

We performed computational simulations of the electrical field distribution induced by tACS based on the simNIBS 2.1^63^. A finite element head model was derived from MRI data of one subject who did not participate in this study. The following parameters were used for this computation: electrode size, 5 × 7 cm; electrode thickness, 1 mm; target electrode position, left-hand knob region in M1: reference electrode position, Pz; current strength, 0.5 mA (1 mA peak-to-peak). The electric field results showed that our stimulation setup covered a relatively large area, including the sensorimotor area (Fig. 1D).

### Procedures

This study had a randomized, cross-over, single-blinded design. Participants underwent three separate sessions that were separated by at least 2 days. rPPS was applied in combination with tACS using three different protocols: rPPS combined with tACS at the 90° phase (peak), rPPS combined with tACS at the 270° phase (trough), and sham tACS (sham) (Fig. 1A). First, we studied the after-effects of β tACS combined with rPPS on M1 (Experiment 1). Then, we explored the after-effects of α tACS combined with rPPS on M1 (Experiment 2). We did not conduct trials in which α and β tACS were delivered alone because it is difficult to blind participants to these conditions. Moreover, a systematic review on tACS revealed that β tACS at a low intensity (≤ 1 mA) over M1 did not modulate MEP amplitudes^57^. rPPS was initiated 60 s after the beginning of the tACS and then continued for 15 min. In the sham protocol, tACS was applied for only 60 s at the beginning of the 16 min period. The TMS and tACS were controlled by PsychoPy^64^. We recorded the tACS signal using a 1 kΩ resistor connected sequentially to a tACS stimulator^21^, which was stored with paired-pulse MEP signals during rPPS. The timing of the TMS pulse with respect to the targeted tACS phase was calculated from the time difference. For instance, if the TMS pulse was synchronized with the phase of the β tACS, the 180° phase difference was calculated as a 25-ms time difference. The relationship between the tACS phase and TMS pulse was monitored online. We investigated the system’s precision across all participants. First, tACS signals were band-pass filtered with a zero-phase FIR filter (Experiment 1: 18–22 Hz; Experiment 2: 8–12 Hz) to reduce noise. Second, we assessed the time delay between the second pulse of the paired-pulse TMS and the target tACS phase (peak or trough). The average time delay during combined stimulation was as follows: 0.5 ± 0.3 ms (SD) (phase lag: 3.7 ± 2.2°) for Experiment 1; 0.7 ± 0.5 ms (phase lag: 2.7 ± 2°) for Experiment 2.

Before the combined stimulation, we collected baseline data in which 12 single-pulse TMS and 12 paired-pulse TMS pulse trains were delivered and single-pulse MEPs and paired-pulse MEPs were recorded, respectively. In the post-intervention period, we recorded 12 single-pulse MEPs every 5 min for up to 30 min after the combined stimulation (Fig. 1B).

### Data analysis and statistics

Data analysis was performed using Python 3.8 (https://www.python.org/) and R 3.6.2^65^ software. We measured peak-to-peak MEP amplitudes. The epochs with muscle activation that took place before the TMS pulse were excluded. The 12 single-pulse MEP as well as 12 paired-pulse MEP amplitudes before the combined stimulation were averaged as the baseline. During the intervention, the amplitudes for every 30 paired-pulse MEPs (T1–T6, a total of 180 paired-pulse MEPs) were averaged and normalized to the baseline of paired-pulse MEPs. After the intervention, 12 single-pulse MEP amplitudes in each block (post 0–30 min) were averaged and normalized to the baseline single-pulse MEPs.

To test the effects of the different conditions after the intervention, we conducted a two-way rmANOVA for the normalized MEP amplitudes with condition (peak, trough, and sham) and time (post 0–30 min) as factors. We studied the effects of different conditions during the intervention using a two-way rmANOVA with condition and time (T1–T6) as main factors. The Huynh–Feldt correction was used when sphericity was lacking. We performed a post-hoc analysis using paired *t*-tests with Bonferroni’s correction for multiple comparisons. To evaluate whether the MEP amplitudes changed from the baseline (pre) to after the combined stimulation, we employed a one-way rmANOVA for the absolute values of MEP amplitude with time (pre, post 0–30 min) as a factor in each condition. For further analyses, Dunnett’s test was applied to determine the time points that were different from the baseline values. Statistical analyses were carried out using SPSS (version 17.0 for Windows, IBM, Armonk, NY, USA).
